# The effect of liver disease on hepatic microenvironment and implications for immune therapy

**DOI:** 10.3389/fphar.2023.1225821

**Published:** 2023-08-07

**Authors:** Zachary J. Brown, Samantha M. Ruff, Timothy M. Pawlik

**Affiliations:** ^1^ Department of Surgery, New York University Long Island School of Medicine, Mineola, NY, United States; ^2^ James Comprehensive Cancer Center, Department of Surgery, The Ohio State University Wexner Medical Center, Columbus, OH, United States

**Keywords:** Hepatocellular carcinoma (HCC), immune microenviroment, immune check inhibitor (ICI), liver disase, non-alcocholic fatty liver disease, cirrhosis, alcohol induced liver disease, viral heaptitis

## Abstract

Hepatocellular carcinoma (HCC) is the most common primary liver cancer and the fourth leading cause of cancer-related death worldwide. HCC often occurs in the setting of chronic liver disease or cirrhosis. Recent evidence has highlighted the importance of the immune microenvironment in the development and progression of HCC, as well as its role in the potential response to therapy. Liver disease such as viral hepatitis, alcohol induced liver disease, and non-alcoholic fatty liver disease is a major risk factor for the development of HCC and has been demonstrated to alter the immune microenvironment. Alterations in the immune microenvironment may markedly influence the response to different therapeutic strategies. As such, research has focused on understanding the complex relationship among tumor cells, immune cells, and the surrounding liver parenchyma to treat HCC more effectively. We herein review the immune microenvironment, as well as the relative effect of liver disease on the immune microenvironment. In addition, we review how changes in the immune microenvironment can lead to therapeutic resistance, as well as highlight future strategies aimed at developing the next-generation of therapies for HCC.

## Introduction

Hepatocellular carcinoma (HCC) is the most common primary liver cancer and the fourth leading cause of cancer-related death worldwide (Forner et al., 2018). Chronic liver disease due to various etiologies such as viral hepatitis, alcohol induced liver disease (ALD), non-alcoholic fatty liver disease (NAFLD), and non-alcoholic steatohepatitis (NASH) is a major risk factor for the development of HCC (Llovet et al., 2016). The severity of the underlying liver disease is often a major factor in determining treatment strategy as it is often a driving factor related to therapeutic morbidity. To this point, patients with advanced tumors or severe underlying liver disease are often not candidates for curative treatment options and these patients are treated with locoregional or systemic therapies (Llovet et al., 2002; Maluccio et al., 2005; Maluccio et al., 2008; Raoul et al., 2019). Recently, there has been increased interest in the use of immune checkpoint inhibitors (ICIs) to treat patient with advanced HCC. To date, response rates and survival related to ICI treatment remain varied and often not durable. As such, there has been increased efforts to understand mechanisms of resistance to ICI therapy.

Recent evidence has highlighted the importance of the liver immune microenvironment in the development and progression of HCC, as well as the potential response to therapy. Research has focused on understanding the complex interactions among tumor cells, immune cells, and the liver tissue. Moreover, there is an emerging understanding as to how the immune microenvironment may change relative to different liver disease etiologies. In addition, there are ongoing efforts to investigate the effect of liver disease on the immune microenvironment, as well as to characterize the impact of liver disease on response to therapy. We herein review the liver immune microenvironment, as well as the impact of liver disease on the immune microenvironment. In addition, we review how changes in the immune microenvironment can lead to therapeutic resistance, as well as highlight future strategies aimed at developing the next-generation of therapies for HCC.

## Overview of the liver immune microenvironment

**FIGURE 1:**
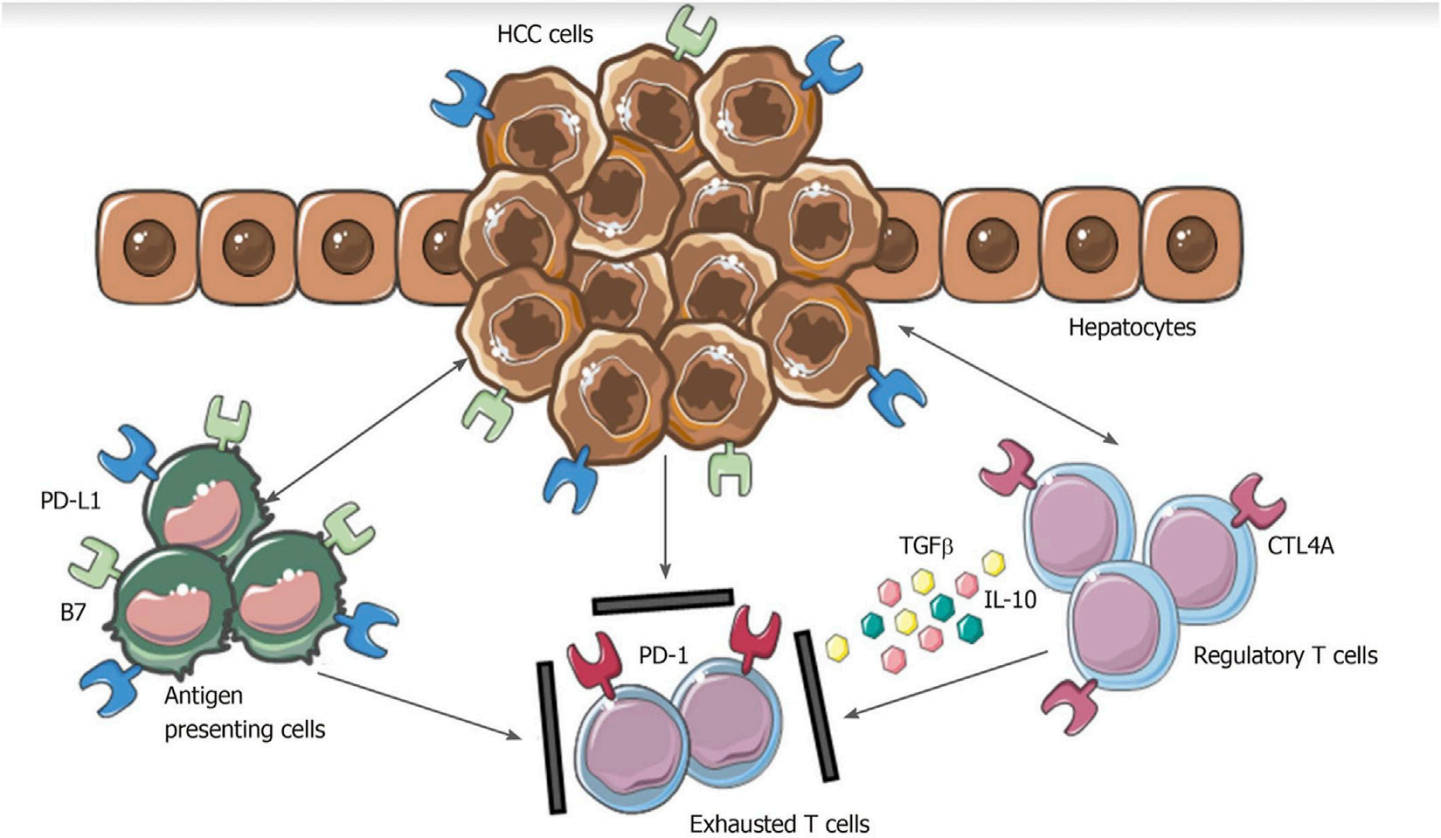
Mechanisms involved in hepatocellular carcinoma immune evasion. In physiological conditions, liver has the ability to induce immunotolerance against antigen from gastrointestinal tract. These mechanisms have a detrimental role during hepatocellular carcinoma development and progression. Upregulation of inhibitory programmed death-ligand 1 molecule from tumor cells, Kupffer cells, liver sinusoidal endothelial cells and antigen presenting cells, together with the release of interleukin-10 and transforming growth factor beta, lead to an exhausted phenotype of CD8^+^ cells and prevent tumor cells from immune damage. HCC: Hepatocellular carcinoma; PD-L1: Programmed death-ligand 1; CTL4A: Cytotoxic T lymphocyte antigen 4; PD-1: Programmed cell death protein 1; TGFβ: Transforming growth factor beta; IL-10: Interleukin-10. From: Polidoro et al. Tumor microenvironment in primary liver tumors: A challenging role of natural killer cells. World J Gastroenterol 2020. PMID 32952338.

**FIGURE 2:**
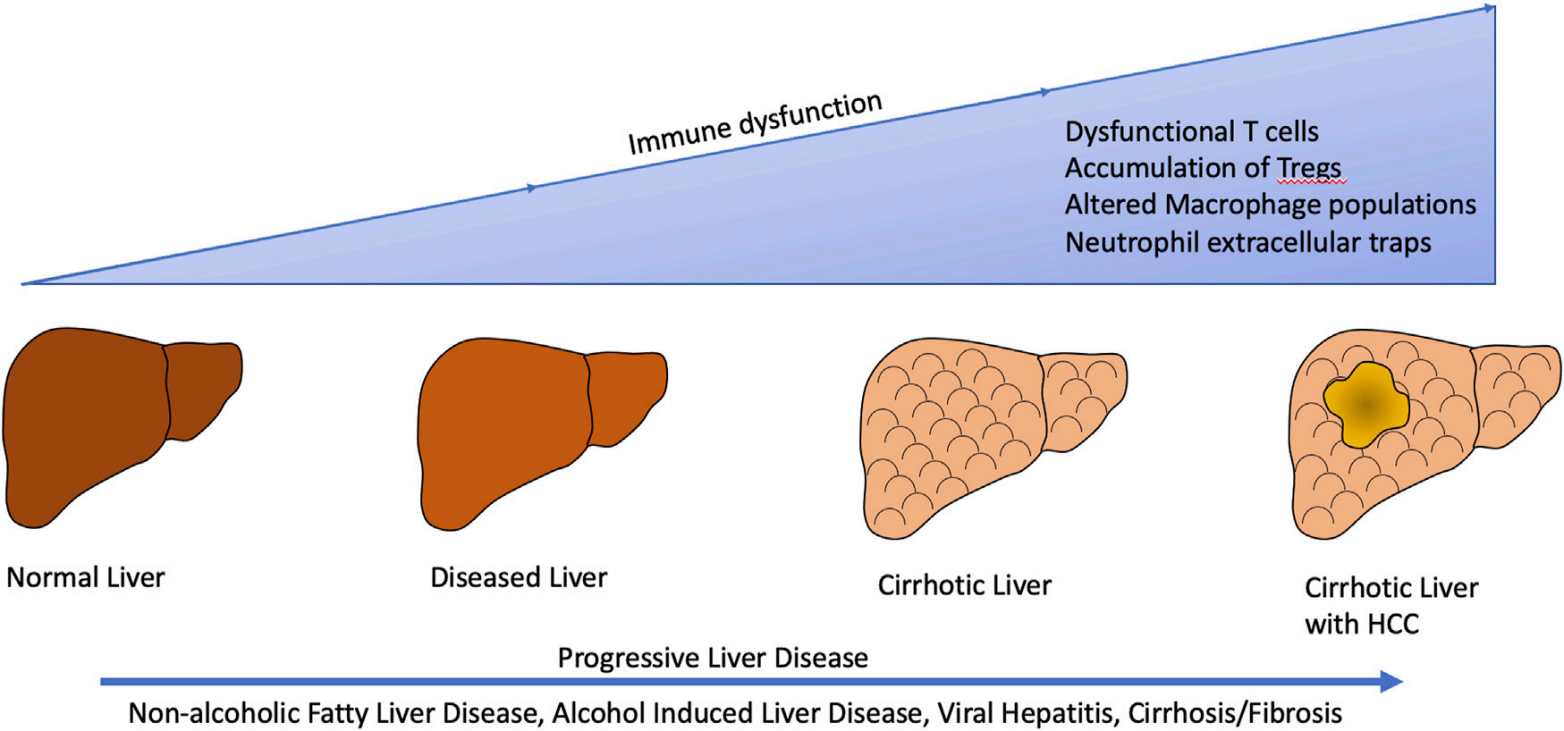
Liver disease from various etiologies such as non-alcoholic fatty liver disease/non-alcoholic steatohepatitis, alcohol induced liver disease, viral hepatitis, and cirrhosis/fibrosis result in alterations in the tumor immune microenvironment subsequently leading to development and progression of hepatocellular carcinoma.

The liver is naturally exposed to a large influx of antigens from the gastrointestinal tract. As such, the liver is uniquely immune tolerant having developed intrinsic tolerogenic mechanisms in the innate and adaptive immune responses (Figure 1). Thus, the liver protects itself from autoimmune damage secondary to large antigen presentation from the gastrointestinal tract (Jenne and Kubes, 2013; Brown et al., 2019). However, the liver also provides a unique proinflammatory microenvironment composed of Kupffer cells, antigen-presenting cells (APCs), T cells, and hepatic stellate cells (HSCs) (Stauffer et al., 2012; Agosti et al., 2018; Koo et al., 2020). During liver injury and disease states, a wide range of liver cells participate in a complex proinflammatory response that can result in hepatocyte death and disease progression (Figure 2). (Koo et al., 2020)

### Innate immune system

In the liver, the innate immune system consists of multiple cell types that act as the first line of defense against pathogens. Kupffer cells (KC) are resident macrophages within the liver, which are in constant contact with antigens arriving to the liver from the gastrointestinal tract (Racanelli and Rehermann, 2006; Nakamoto and Kanai, 2014; Tacke, 2017). In turn, the KC serve as the first line of immune defense. Additionally, a large population of peripheral monocytes are often recruited to the liver. Kupffer cells can be distinguished from monocyte derived macrophages as KCs have low levels of CD11b and CCR2, and high F4/80 expression (Holt et al., 2008; Obstfeld et al., 2010; Stienstra et al., 2010). Furthermore, Bleriot et al. identified two distict populations of KCs which shared a core molecular signature while expressing different genes and proteins (Blériot et al., 2021). Similarly, macrophages exists in multiple subtypes such as the M1 phenotype with antitumor inflammatory reactions and the M2 phenotype characterized by tumor promoting capabilities with immune suppression (Liu et al., 2021).

Natural killer (NK) cells are another subset of the innate immune system that have cytolytic activity against stressed cells, virally infected cells, and malignant cells (Abul et al., 2007; Kahraman et al., 2010). Unlike CD8^+^ T-cells, which require costimulation for cytotoxic activity, NK cells have the unique ability to kill targeted cells without a need for secondary activation. Neutrophils, the most abundant population of circulating white blood cells, activate early phases of the inflammatory response in the innate immune system (Abul et al., 2007). Dendritic cells (DCs), a type of APC, are innate immune cells that present antigens to T-cells thus initiating the adaptive immune response (Abul et al., 2007).

### Adaptive immune system

Recent evidence has highlighted the changes in the adaptive immune system in the immune microenvironment secondary to liver disease. T-cells are abundant in healthy livers and exist in several subsets: CD4^+^ helper T (Th) cells, CD8^+^ cytotoxic T cells, and regulatory T-cells (Tregs) (Ramadori et al., 2022). CD4^+^ T cells are essential for tumor control to prevent tumor initiation and facilitate clearance of premalignant and malignant cells (Rakhra et al., 2010; Kang et al., 2011; Heinrich et al., 2021). CD4^+^ T cells are often initiators of an anti-tumor response and are associated with a favorable response to immunotherapy. CD8^+^ cytotoxic T-cells are the main effector cells of the cellular immune system and eliminate infected or malignant cells through recognition of presented antigens (Van Herck et al., 2019). Additionally, there is a population of CD8^+^ tissue-resident memory (TRM) cells that reside in the liver and act as local immune sentinels (MacParland et al., 2018; Hirsova et al., 2021).

While CD4^+^ and CD8^+^ T cells promote an anti-tumor inflammatory response, Tregs are an immunosuppressive subset of CD4^+^ T-cells and are essential to maintain homeostasis and immune tolerance (Togashi et al., 2019; Wang et al., 2021). The accumulation of Tregs has been recognized as promoting immune evasion and hepatocarcinogenesis (Togashi et al., 2019; Wang et al., 2021). Natural killer T cells (NKT) are considered a bridge between innate and adaptive immunity via expression of NK cell surface markers as well as antigen receptor characteristics of T-cells (Abul et al., 2007; Arrese et al., 2016). NKT cells are located in the sinusoids of the liver to provide intravascular immune surveillance (Geissmann et al., 2005; Abul et al., 2007). NKT cells are both proinflammatory mediated through the type I NKT cell subtype, as well as immune suppressive protecting against liver injury via Type II NKT cells (Kumar, 2013).

## Influence of liver disease on the hepatic immune microenvironment

### Non-alcoholic fatty liver disease

Non-alcoholic fatty liver disease (NAFLD) and its severe form non-alcoholic steatohepatitis (NASH) are characterized by the accumulation of triglycerides within hepatocytes with approximately 10%–20% of patients progressing to cirrhosis (Cusi, 2012). NAFLD and NASH are a manifestation of metabolic syndrome, which is generally characterized as a constellation of type 2 diabetes mellitus, dyslipidemia, obesity, and cardiovascular disease (Cusi, 2012). In the United States, the prevalence of NASH is increasing and is becoming a significant risk factor for the development of HCC (Sheka et al., 2020).

NAFLD/NASH have multiple effects on the immune microenvironment. In the innate immune system, CCR2 macrophages are increased in the liver correlating with levels of CCL2 found in steatotic hepatocytes (Obstfeld et al., 2010; Stienstra et al., 2010). In preclinical studies, drugs targeting the CCL2/CCR2 axis impaired macrophage recruitment to the liver and reduced hepatosteatosis, inflammation, and fibrosis (Baeck et al., 2012; Lefebvre et al., 2016). In addition, there is often a large influx of neutrophils among patients with NASH (Feng et al., 2011). Myeloperoxidase (MPO) is used by neutrophils to create reactive oxygen species (ROS) in order to kill microbes. In patients with NASH, MPO is often increased suggesting that accumulation of MPO and ROS contribute to the development of NASH (Rensen et al., 2009). Neutrophils also exacerbate liver inflammation through the recruitment of macrophages and APCs (Arrese et al., 2016). Additionally, neutrophils release neutrophil extracellular traps (NETs), which are long chromatin fibers embedded with inflammatory proteins and neutrophil proteases (van der Windt et al., 2018; Wang et al., 2021). Preclinical studies suggest NET formation in the early stages NAFLD and increases with the progress to NASH (Wang et al., 2021).

Relative to the adaptive immune response, liver biopsies among patients with NASH have demonstrated increased infiltrating clusters of B cells and T cells that correlate with increased levels of oxidative stress-derived epitopes released from damaged hepatocytes (Garnelo et al., 2017; Sutti and Albano, 2020). Additionally, preclinical studies indicate ROS-dependent cell death of hepatic CD4^+^ T cells can occur leading to impaired anti-tumor surveillance (Ma et al., 2016; Brown et al., 2018). Furthermore, in early stages of NASH, there is an obesity-induced hepatic type I interferon (INF-1) response that has been associated with increased pathogenic CD8^+^ T-cell production of proinflammatory cytokines, which contributes to hepatocyte damage (Ghazarian et al., 2017). Of note, several investigators have reported improvement of NASH and restored hepatic insulin sensitivity and reduced fibrosis using experimental models with deletion of CD8^+^ T cells (Ghazarian et al., 2017; Van Herck et al., 2019). NKT cells also contribute to both the development and progression of NASH (Syn et al., 2010). Patients with NASH cirrhosis have four times as many NKT cells than individuals with healthy livers (Syn et al., 2012). Wolf et al. reported cross-talk between CD8 T-cells, NKT cells, and hepatocytes in the setting of NASH development and transition to HCC (Wolf et al., 2014). These investigators reported that experimental reduction of NKT cells, despite elevated CD8^+^ T cells, prevented liver damage. In turn, the data suggested that CD8 T-cells alone are not sufficient to cause liver damage in the absence of NKT cells (Wolf et al., 2014).

In addition to changes in pro-inflammatory cells within the immune microenvironment, alterations in immunosuppressive Tregs have been noted. In one experimental mouse model, Tregs were noted to be increased in NASH-livers with a lower concentration of CD4^+^ T cells (Wang et al., 2021). When Tregs were depleted, HCC initiation and progression of NASH was drastically inhibited (Wang et al., 2021). Furthermore, an imbalance between helper T cells and Tregs can promote progression of NAFLD along with higher expression of inflammatory cytokines (He et al., 2017; Zhang CY. et al., 2022).

### Viral hepatitis

Viral hepatitis is the leading cause of HCC worldwide (D'Souza et al., 2020). Similar to NAFLD/NASH, chronic liver disease caused by viral hepatitis has an effect on the immune microenvironment. A study by DeBattista et al. demonstrated that hepatitis B viral (HBV) and hepatitis C virus (HCV) have distinct molecular signatures and immune landscapes within the liver (De Battista et al., 2021). For example, among patients with HBV-HCC, there was a lower proportion of differentially expressed genes related to the immune response, yet a higher number of upregulated genes *versus* patients with HCV-HCC. In addition, HCV-HCC was characterized by downregulation of immune genes within the tumor especially related to T-cells, as well as upregulation of oxidative stress genes (De Battista et al., 2021). In contrast, the molecular signature of HBV-HCC was characterized by the upregulation of genes related to cell cycle control and monocyte/macrophage activation (De Battista et al., 2021).

Non-viral related HCC and HBV-HCC immune microenvironment appear to be composed of distinct immune subsets. Lim et al. utilized cytometry by time of flight (CyTOF) to perform in-depth immunoprofiling and reported that the HBV-HCC immune microenvironment was more immunosuppressive and exhausted with increased Tregs and CD8^+^ resident memory T cells (Lim et al., 2019). Increased Treg were associated with a poor prognosis, while CD8^+^ resident memory T cells were associated with a favorable prognosis (Lim et al., 2019). In a separate study, Li et al. reported that patients with higher levels of Tregs in the peripheral blood and/or tumor sites had a worse prognosis (Li et al., 2016). In pre-clinical mouse models, depleting Tregs was potentially therapeutic for HBV-related liver diseases through induction of antiviral and antitumor immunity. In turn, the data suggested that Tregs play a role in the development of cirrhosis, the transformation of cirrhosis to HCC, and the progression and metastasis of HCC (Li et al., 2016). In yet another study, Zhang et al. demonstrated that HBV-HCC, HCV-HCC and non-viral HCC had similar molecular phenotypes with inhibition of immune pathways. In the immune microenvironment associated with virus induced HCC there was, however, excessive M2-type macrophage polarization associated with immune suppression (Zhang YZ. et al., 2022). Similarly, Ding et al. performed a meta-analysis of 1,520 patients and noted that infiltration of immune cells in the tumor microenvironment for viral associated HCC *versus* non-viral associated HCC differed relative to M0 macrophages, M2 macrophages, Tregs, naive B cells, follicular helper T cells, activated dendritic cells, activated mast cells, and plasma cells (Ding et al., 2021).

The influence of viral hepatitis on the development and progression of HCC is complex and may initially be benefitial recruiting immune cells to protect against HCC development (Zamor et al., 2017). Among patients with HCV, medications such as direct acting antivirals (DAAs) are used to eradicate the virus from infected individuals. Recent investigators have focused on the effect that DAAs may have on HCC tumorigenesis after eradication of viral hepatitis. Reports have described early occurance and recurrence of HCC in patients who where successfully treated with DAAs (Conti et al., 2016; Reig et al., 2016). With DAA therapies for HCV infection, it is common to see a sustained virological response (SVR). However, reactivation of HBV in patients with co-infection and development of HCC among patients who achieved SVR has been observed (Borgia et al., 2021). It has been hypothesized that changes occur in intrahepatic immune surveillance following a SVR. Amaddeo et al. evaluate changes in the immune microenvironment after HCV eradication by comparing patients with HCC treated with DAA who had a SVR *versus* untreated controls (Amaddeo et al., 2020). Interestingly, there was no difference in immune profiles between the two groups, but there was a down regulation of interferon related genes after DAA treatment (Amaddeo et al., 2020). More studies are required to understand the effect of DAAs on the immune microenvironment, as well as the pathogenesis of HCC development of HCC among patients with a SVR.

### Cirrhosis/fibrosis

Most HCC tumors arise in the setting of chronic liver disease and liver cirrhosis/fibrosis, which has a dramatic effect on the immune microenvironment. Ke et al. investigated the role of liver fibrosis to regulate tumor-infiltrating lymphocytes (TILs) and induce immunosuppression (Ke et al., 2021). Among patients with HCC, high CD8^+^ T cell infiltration was correlated with prolonged survival (Ke et al., 2021). Indeed, in mouse models with CCl_4_-induced liver fibrosis, as well as fibrotic human livers, elevated expression of immune checkpoints and decreased antitumor immunity was noted *versus* the control group (Ke et al., 2021). In addition, compared with patients who had low fibrosis scores, patients with high fibrosis scores had a significant reduction in tumor-cell-killing capacity of NK cells (Amer et al., 2018). Furthermore, in preclinical studies, Brandt et al. investigated the chemokine CXCL10 during fibrosis-associated hepatocarcinogenesis (Brandt et al., 2022). Of note, mice with *Cxcl10* deficiency exhibited attenuated hepatocarcinogenesis. When fibrosis was induced, there was a pro-inflammatory tumor microenvironment, an accumulation of anti-tumoral immune cells in the tissue, and an accumulation of anti-tumoral T cells in the invasive tumor margin (Brandt et al., 2022).

### Alcohol induced liver disease

Alcohol induced liver disease remains a major risk factor for the development of HCC contributing to nearly 30% of cases (McKillop and Schrum, 2009; Akinyemiju et al., 2017). Alcohol induced liver disease is a spectrum encompassing fatty liver, alcoholic hepatitis, and cirrhosis (Singal et al., 2014; Jinjuvadia et al., 2015). With chronic alcohol consumption, there is induction of the enzyme CYP2E1 which becomes the primary pathway of alcohol metabolism rather than alcohol dehydrogenase (Lu and Cederbaum, 2008). As a result of altered metabolism, there is increased acetaldehyde which carries metagenic and carcinogenic properties (Brooks and Theruvathu, 2005; McKillop and Schrum, 2009). Alcohol consumption has also been shown to cause alterations in the gut microbiome with increased absorption of endotoxin leading to activation of KCs (Bajaj et al., 2014). This activation of KCs results in the release of inflammatory cytokines causing increased collagen deposition, scarring and ultimately fibrosis (Thurman, 1998; Bode and Bode, 2005; Nagata et al., 2007). Furthermore, preclinical studies suggest chronic alcohol consumption reduces Tregs and causes an increase in helper T cells (Chen et al., 2016). The molecular mechanisms and full impact of changes in immune subsets on the progression of alcoholic liver disease has not yet been fully elucidated (Zhang CY. et al., 2022).

## Immune checkpoint inhibitors and HCC

**FIGURE 3:**
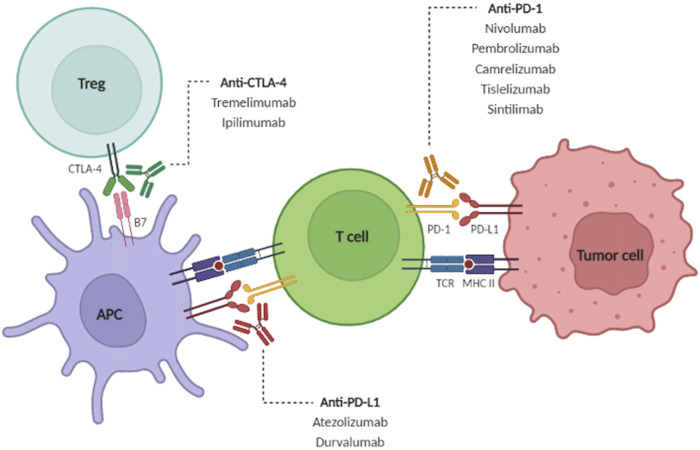
Under physiologic conditions, tumor antigens are recognized and presented to CD4^+^ T cells, which in turn further activate CD8^+^ T cells to initiate immune attack. T cell activation causes upregulation of CTLA-4 and PD-1 to prevent overactivation of the immune response. Immune checkpoint inhibitors block these inhibitory signals to increase the anti-tumor immune response. From: Brown ZJ et al. Safety, efficacy, and tolerability of immune checkpoint inhibitors in the treatment of hepatocellular carcinoma. Surgical Oncology June 2022. PMID 35395582.

ICIs are now a therapeutic option for many malignancies and indications continue to expand (Le et al., 2015; Le et al., 2017; Eso et al., 2020). Inflammation plays a central role in the development of HCC as it drives carcinogenesis and therefore immunotherapies, including ICIs, have been proposed as part of an ideal treatment strategy for patients with HCC (Jenne and Kubes, 2013; Makarova-Rusher et al., 2015). With chronic antigen exposure, programmed cell death-1 (PD-1) is unregulated on immune cells including CD4^+^ and CD8^+^ T cells, NK cells, B cells, monocytes, DC, as well as immunosuppressive cells such as Tregs and myeloid-derived suppressor cells (MDSCs) (Prieto et al., 2015). When PD-1 binds with its ligand, PD-L1 and PD-L2, T cell receptor signaling is inhibited and thereby creates an exhausted dysfunctional T cell phenotype (Prieto et al., 2015) **(** Figure 3). Cancer cells have utilized this mechanism to form an immunosuppressive microenvironment allowing tumors cells to be unchecked by the immune system (Prieto et al., 2015). In addition, activation of T cells upregulates the immunosuppressive receptor, cytotoxic T lymphocyte associated protein-4 (CTLA-4). CTLA-4 acts as a check on the adaptive immune response by taking away the necessary costimulatory signal for T cell activation (Figure 3). CTLA-4 is present on activated T cells, DCs, and constitutively expressed on Tregs (Prieto et al., 2015; Inarrairaegui et al., 2017). Drugs targeting the PD-1/PD-L1 and CTLA-4/CD80/CD86 axes alone or in combination have been reported to be safe and effective among patients with advanced HCC (Figure 3). In addition, new and novel combination therapies are being tested (Table 1).

**TABLE 1:** Ongoing studies of combination therapies with immune checkpoint inhibitors for patients with hepatocellular carcinoma.

NCT number	Title	Intervention	Characteristics	Enrollment	Location
NCT04826406	A Study of Camrelizumab Combined Apatinib in Hepatocellular Carcinoma Previously Treated With Immune Checkpoint Inhibitors	Camrelizumab	Phase 2	40	China
		Apatinib			
NCT04696055	Regorafenib Plus Pembrolizumab in Patients With Advanced or Spreading	Pembrolizumab	Phase 2	95	International
	Liver Cancer Who Have Been Previously Treated With PD-1/PD-L1 Immune Checkpoint Inhibitors	Regorafenib			
NCT03970616	A Study of Tivozanib in Combination with Durvalumab in Subjects With Advanced HCC	Tivozanib	Phase 1	42	United States
DEDUCTIVE		Durvalumab	Phase 2		
NCT05178043	GT90001 Plus Nivolumab in Patients With Advanced HCC	Nivolumab	Phase 2	105	United States
		GT90001			
NCT05048017	Regorafenib Combined With PD-1 Inhibitor Therapy for Secondline Treatment of HCC	Regorafenib	Phase 2	20	China
		PD-1 inhibitor			
NCT04183088	Regorafenib Plus Tislelizumab as First-line Systemic Therapy for Patients With Advanced HCC	Tislelizumab regorafenib	Phase 2	125	Taiwan
NCT05086692	A Beta-only IL-2 ImmunoTherapY Study	MDNA11	Phase 1	100	International
ABILITY		ICI	Phase 2		
NCT04050462	Nivolumab Combined With BMS-986253 in HCC Patients	Nivolumab	Phase 2	23	United States
		Cabiralizumab			
		BMS-986253			
NCT03893695	Combination of GT90001 and Nivolumab in Patients With Metastatic HCC	GT90001 and Nivolumab	Phase 1	20	Taiwan
			Phase 2		
NCT03682276	Safety and Bioactivity of Ipilimumab and Nivolumab Combination Prior to Liver Resection in HCC	Ipilimumab	Phase 1	32	United Kingdom
PRIME-HCC		Nivolumab	Phase 2		
NCT05257590	CVM-1118 in Combination With Nivolumab for Unresectable Advanced HCC	Nivolumab	Phase 2	95	Taiwan
		CVM-1118			
NCT04567615	A Study of Relatlimab in Combination With Nivolumab in Participants With Advanced Liver Cancer Who Have Never Been Treated With Immuno-oncology Therapy After Prior Treatment With Tyrosine Kinase Inhibitors	Nivolumab	Phase 2	250	International
		Relatlimab			
NCT03841201	Immunotherapy With Nivolumab in Combination With Lenvatinib for Advanced Stage HCC	Lenvatinib	Phase 2	50	Germany
		Nivolumab			
NCT01658878	An Immuno-therapy Study to Evaluate the Effectiveness, Safety and Tolerability of Nivolumab or Nivolumab in Combination With Other Agents in Patients With Advanced Liver Cancer	Nivolumab	Phase 1	659	International
		Sorafenib			
CheckMate040		Ipilimumab	Phase 2		
		Cabozantinib			
NCT04039607	A Study of Nivolumab in Combination With Ipilimumab in Participants With Advanced HCC	Nivolumab	Phase 3	732	International
CheckMate9DW		Ipilimumab			
		Sorafenib lenvatinib			
NCT04170556	Regorafenib Followed by Nivolumab in Patients With HCC	Regorafenib	Phase 1	78	Spain
GOING		Nivolumab	Phase 2		
NCT03539822	Cabozantinib Plus Durvalumab With or Without Tremelimumab in Patients	Cabozantinib	Phase 1	117	United States
CAMILLA	With Gastroesophageal Cancer and Other Gastrointestinal Malignancies	Durvalumab Tremelimumab	Phase 2		
NCT04102098	A Study of Atezolizumab Plus Bevacizumab Versus Active Surveillance as Adjuvant Therapy in Patients with HCC at High Risk of Recurrence After Surgical Resection or Ablation	Atezolizumab Bevacizumab	Phase 3	668	International
IMbrave050					
NCT04912765	Neoantigen Dendritic Cell Vaccine and Nivolumab in HCC and Liver Metastases From CRC	Neoantigen	Phase 2	60	Singapore
		Dendritic Cell Vaccine			
		Nivolumab			
NCT03829436	TPST-1120 as Monotherapy and in Combination With Nivolumab in Subjects With Advanced Cancers	TPST-1120 nivolumab	Phase 1	138	United States
NCT03170960	Study of Cabozantinib in Combination With Atezolizumab to Subjects With Locally Advanced or Metastatic Solid Tumors	cabozantinib	Phase 1	1732	International
		atezolizumab	Phase 2		
NCT05176483	Study of XL092 in Combination With Immuno-Oncology Agents in Subjects With Solid Tumors	XL092	Phase 1	1,078	International
		Nivolumab			
		Ipilimumab			
		Relatlimab			
NCT05337137	A Study of Nivolumab and Relatlimab in Combination With Bevacizumab in Advanced Liver Cancer	Relatlimab	Phase 1	162	International
RELATIVITY-106		Nivolumab	Phase 2		
		Bevacizumab			
NCT03439891	Sorafenib and Nivolumab in Treating Participants With Unresectable, Locally Advanced or Metastatic Liver Cancer	Nivolumab	Phase 2	16	United States
		Sorafenib			

Abbreviations: HCC, hepatocellular carcinoma; ICI, immune checkpoint inhibitor.

### Atezolizumab

Atezolizumab is a PD-L1 inhibitor that is now standard first line therapy in combination with bevacizumab (atezo-bev) for patients with advanced HCC based on the IMbrave150 trial (Finn et al., 2020a). Among patients with advanced HCC, atezo-bev demonstrated a 12 month overall survival (OS) of 67.2% *versus* 54.6% for patients in the sorafenib cohort; median progression free survival (PFS) was 6.8 *versus* 4.3 months in the atezo-bev and sorafenib cohorts, respectively. Of note, this trial only included patients with preserved liver function and therefore may not be applicable to the large population of patients in which HCC arises in the setting of liver dysfunction. Real world retrospective studies have compared atezo-bev to sorafenib or lenvatinib among patients with advanced HCC and liver dysfunction (Kim et al., 2022; Hiraoka et al., 2023; Jost-Brinkmann et al., 2023; Lee et al., 2023). These studies have demonstrated a similar survival advantage in the atezo-bev cohort (Kim et al., 2022; Hiraoka et al., 2023; Jost-Brinkmann et al., 2023; Lee et al., 2023). Currently, the IMbrave050 trial is evaluating the efficacy of adjuvant atezo-bev *versus* surveillance among patients with resected or ablated HCC (NCT04102098).

### Tremelimumab and durvalumab

Tremelimumab, a CTLA-4 inhibitor, has had limited efficacy as monotherapy in preliminary clinical trials; in turn, combination therapy with the PD-L1 inhibitor, durvalumab, has been investigated (Sangro et al., 2013). In a phase II randomized trial, patients with advanced HCC received various combinations of tremelimumab and durvalumab or either drug as monotherapy (Kelley et al., 2021). The greatest efficacy was noted among patients treated with a tremelimumab priming dose and 4 weeks of durvalumab, resulting in an ORR of 24% and median OS of 18 months (Abou-Alfa et al., 2022). The follow-up phase III HIMALAYA trial compared durvalumab monotherapy, sorafenib, or a priming dose of tremelimumab with weekly durvalumab among patients who were treatment naïve with advanced HCC (Abou-Alfa et al., 2022). This study demonstrated that combination tremelimumab/durvalumab resulted in a median OS of 16.4 months *versus* 13.8 months among patients in the sorafenib cohort. As a result of this trial, combination tremelimumab/durvalumab was approved for patients with unresectable HCC in the United States and Europe (Keam, 2023). There is currently an ongoing phase III trial (EMERALD-3, NCT05301842) for patients with locally advanced HCC not amenable to curative transplant, ablation, or surgery. Patients are randomized to receive either the combination of transarterial chemoembolization (TACE), durvalumab, and tremelimumab with or without lenvatinib *versus* TACE alone.

### Nivolumab and ipilimumab

Nivolumab is a PD-1 inhibitor first approved as second-line therapy for HCC. The Checkmate 040 trial evaluated nivolumab among patients with advanced HCC (up to Child-Pugh B) who may or may not have been treated with sorafenib (Kudo et al., 2021). The median duration of response was 9.9 months with an ORR of 12% and disease control rate of 55%. Nivolumab had an acceptable safety profile, including patients with underlying liver disease. The Checkmate 459 trial compared nivolumab with sorafenib among patients with advanced HCC in the first line setting (Yau et al., 2022). While there was no significant difference in OS between the two treatment arms, the results are difficult to interpret because several patients crossed over to the nivolumab arm after progressing on sorafenib. Retrospective studies have demonstrated similar findings with no survival advantage seen with nivolumab over sorafenib (Chapin et al., 2023).

As single agent, nivolumab demonstrated no improvement is survival compared with sorafenib; the combination of nivolumab and ipilimumab (CTLA-4 inhibitor) was administered at different doses and intervals to patients with advanced HCC previously treated with sorafenib (Yau et al., 2022). At 24 months, the OS for the combination nivolumab/ipilimumab cohort was 40%. Currently, a phase II randomized trial evaluating neoadjuvant nivolumab *versus* nivolumab/ipilimumab for patients with resectable HCC is in process (NCT03222076). Pre-liminary data has demonstrated a median PFS of 19.5 months for the nivolumab/ipilimumab cohort *versus* 9.4 months in the nivolumab monotherapy cohort (Kaseb et al., 2022). Several other ongoing trials are investigating the use of ICIs in the neoadjuvant and adjuvant setting (NCT 03682276, NCT 03299946, Checkmate 9DX, NCT 03383458).

### Pembrolizumab

Pembrolizumab is a PD-1 inhibitor and has had limited success in clinical trials as a single agent therapy for HCC (Zhu et al., 2018; Finn et al., 2020b). These data have resulted in other trials investigating the combination of pembrolizumab and lenvatinib (tyrosine kinase inhibitor), which has demonstrated a median PFS of 9.3 months and median OS of 22 months in phase I trial of patients with advanced HCC (Finn et al., 2020c). In a different study, Chen et al. reported on 170 treatment-naïve patients with unresectable HCC treated with the combination of pembrolizumab and lenvatinib with or without a hepatic artery infusion pump (HAIP) (Chen et al., 2021). Median OS was 17.7 months was in the HAIP/pembrolizumab/lenvatinib cohort *versus* 12.6 months among patients in the pembrolizumab/lenvatinib cohort (Chen et al., 2021). Currently, the LEAP-012 phase III randomized clinical trial is evaluating the use of TACE with or without pembrolizumab/lenvatinib for patients with intermediate stage HCC (NCT04246177).

### Mechanisms of resistance and influence of the immune microenvironment

Although there has been success in treatment of patient with advanced HCC using ICIs, response rates remain variable, sometimes poor, and often not durable. Mechanisms of resistance to immune therapies are becoming increasingly understood and this information may lead to improvement in outcomes through better patient selection or more targeted combination therapies. In general, there are two types of resistance to ICIs: primary and secondary/acquired. Primary resistance is characterized by failure of the HCC tumor to respond initially to ICIs. As evidenced in clinical trials, ICIs are only effective in about 30%–40% of patients with HCC, likely due to primary resistance (De Lorenzo et al., 2022). There are several mechanisms of primary resistance. One theory is related to the tumor mutational burden (TMB). A high TMB results in more neoantigens and possibly increased immune recognition, thereby making the tumor more immunogenic. Data from several studies have compared patients with low *versus* high TMB, have noted improved OS with ICIs in the latter group of individuals (Rizvi et al., 2015; Van Allen et al., 2015; Hugo et al., 2016; Ang et al., 2019). Another mechanism of primary resistance is dysfunctional neo-antigen presentation either through acquired genetic mutations that alter antigen presentation or decrease neo-antigen expression (McGranahan et al., 2016; McGranahan et al., 2017; Chowell et al., 2018; Ichinokawa et al., 2019). HCC tumors often contain a high copy number alteration burden and commonly have chromosome instability leading to a loss of genes needed for antigen presentation (Bassaganyas et al., 2020). To support this theory, Haber et al. demonstrated that patients with HCC who had upregulation of MHC-II molecules and increased neo-antigen presentation had a better response to ICIs (Haber et al., 2023).

Recent efforts have focused on the impact of liver disease on the immune microenvironment and subsequent response to therapy. For example, in a subgroup analysis of patients from IMBrave150 that evaluated atezo-bev, the ORR among patients with NASH-related HCC was 27% *versus* 35% among patients with HCC due to other etiologies (Ducreux et al., 2021). Pre-clinical studies have demonstrated loss of CD4^+^ T-cells in association with NASH suggesting immunotherapy may be impaired in the setting of NASH related hepatic tumors. Additionally, steatohepatitis was noted to reduce the ability of immunotherapeutic agents thereby inhibiting hepatic tumor growth through reduction of tumor infiltration by CD4^+^ T cells and effector memory cells (Ma et al., 2016; Brown et al., 2018; Heinrich et al., 2021).

Secondary or acquired resistance is characterized by patients who have disease recurrence or progression after initially responding to ICIs (De Lorenzo et al., 2022). These mechanisms are poorly understood, but are likely driven by tumor heterogeneity. While PD-1/PD-L1 and CTLA-4 are the more commonly targeted immune checkpoints, other immune checkpoints exist and their presence in the immune microenvironment may impact response to therapy. Targeting additional immune checkpoints, like TIM-3 or LAG-3, using combination therapy may help overcome immune exhaustion and secondary resistance (Zhou et al., 2017). In addition, tumor heterogeneity often results in ICI-sensitive and ICI-resistant cells. In theory, these resistant cells can survive after ICI therapy and clone themselves to become the majority population within the tumor. This process may explain why some patients respond to ICIs, but then ultimately progress (Weiss and Sznol, 2021). Profiling the tumor and using combination therapy may allow us to overcome tumor heterogeneity.

Epigenetics regulate gene expression without altering the DNA sequence. Alterations of epigenomic drivers can promote cancer onset, progression, and influence response to chemotherapy (Hogg et al., 2020; Wu et al., 2021). A study by Wu et al. demonstrated that patients with high epigenetic related genes (ERGs) benefited more from ICIs whereas patients with low ERGs had more T cell dysfunction and subsequentlyless clinical benefit from ICIs (Wu et al., 2021). In addition, the use of next-generation sequencing (NGS) has been utilized to determine predictive and prognostic information. Using NGS, Harding et al. found that patients with HCC tumors harboring *Wnt/*CTNNB1 mutations were refractory to ICIs with an associated shorter disease control rate, PFS, and OS (Harding et al., 2019).

Liver transplantation (LT) is the preferred treatment strategy for patients with liver cirrhosis and HCC as LT treats both the malignancy, as well as the underlying liver disease (Brown et al., 2023). Traditional LT criteria limit the potential pool of candidates based on strict HCC size and number (Milan criteria: 1 tumor >5 cm; 3 or fewer > 3 cm) (Adam et al., 2018). More recent data have demonstrated that patients successfully down-staged to within Milan LT criteria have post-transplant results similar to patients who initially present within Milan criteria (Yao et al., 2015; Kardashian et al., 2020). While ICIs have changed the treatment paradigm for patients with HCC, ICIs have only been sparingly used in the field of LT due to the potentially fatal complication of allograft rejection (Takamoto et al., 2023). Of note, graft rejection has been reported to be as high as 45% when ICIs are given prior to LT, especially if ICIs are administered within 90 days of LT (Qiao et al., 2021; Schnickel et al., 2022). Other studies have reported using ICIs for downstaging prior to LT, noting it to be relatively safe with a rejection rate of approximately 25% (Tabrizian et al., 2021; Gu et al., 2023). Kuo et al. investigated the washout period between last ICI dose and LT and noted a 42 days washout period for atezolizumab, nivolumab, or pembrolizumab (Kuo et al., 2023).

Several studies have also reported using ICIs following LT to prevent tumor recurrence with a rejection rate of 18.5% (Gu et al., 2023). Interestingly, Rudolph et al. noted that receipt of ICIs 3 months prior to LT may be safer than post-LT ICI administration (Rudolph et al., 2023). A current clinical trial (NCT0518550) is investigating atezo/bev in combinaton with TACE prior to LT among patients with HCC beyond Milan Crietria. The goal of the study is to assess the possibility to downstage patients and not increase the risk of 1-year post-transplant rejection. More data are needed to define the role of ICIs among patient undergoing LT patients. In particular, the competing mechanisms of anti-rejection medications and ICIs on the immune microenviroment require further elucidation.

## Conclusion

Immune checkpoint inhibitors have been adopted as first line therapy for patients with advanced HCC. However, response rates remain variable and a majority of patients do not receive clinical benefit from ICI therapy. Recent efforts have focused on mechanisms of resistance to understand better why patients fail to response to ICIs. The immune microenvironment is frequently altered by liver disease, which can influence patient response to ICI treatment. A better understanding of the influence liver disease has on the immune microenvironment combined with knowledge gained from NGS and epigenetic alterations may improve patient selection, as well as provide novel targeted therapies to improve tumor response. In particular, the ability to understand and successfully target escape pathways may lead to improved outcomes for patients with advanced HCC.
